# Responses of chimpanzees to cues of conspecific observation^☆^

**DOI:** 10.1016/j.anbehav.2013.06.015

**Published:** 2013-09

**Authors:** Daniel Nettle, Katherine A. Cronin, Melissa Bateson

**Affiliations:** a Centre for Behaviour and Evolution & Institute of Neuroscience, Newcastle University, Newcastle, U.K.; b Comparative Cognitive Anthropology Group, Max Planck Institute for Psycholinguistics & Max Planck Institute for Evolutionary Anthropology, Nijmegen, Netherlands

**Keywords:** chimpanzee, cooperation, *Pan troglodytes*, reputation, social intelligence, watching eyes

## Abstract

Recent evidence has shown that humans are remarkably sensitive to artificial cues of conspecific observation when making decisions with potential social consequences. Whether similar effects are found in other great apes has not yet been investigated. We carried out two experiments in which individual chimpanzees, *Pan troglodytes*, took items of food from an array in the presence of either an image of a large conspecific face or a scrambled control image. In experiment 1 we compared three versions of the face image varying in size and the amount of the face displayed. In experiment 2 we compared a fourth variant of the image with more prominent coloured eyes displayed closer to the focal chimpanzee. The chimpanzees did not look at the face images significantly more than at the control images in either experiment. Although there were trends for some individuals in each experiment to be slower to take high-value food items in the face conditions, these were not consistent or robust. We suggest that the extreme human sensitivity to cues of potential conspecific observation may not be shared with chimpanzees.

## Highlights

• Humans respond strongly to ‘watching eyes’, but whether chimpanzees do is unknown.
• We investigated if chimpanzees' readiness to take food was affected by face images.
• There was no clear evidence that subjects looked at faces more than control images.
• Any effect of the faces on chimpanzees' decisions was weak and inconsistent.
• Chimpanzees may not be as sensitive to cues of being watched as humans are.

A number of recent studies have shown that humans are remarkably sensitive to cues of conspecific observation when making decisions with potential social consequences. When images of ‘watching eyes’ are displayed, people are more reluctant to take an available resource for themselves (Burnham 2003; Haley & Fessler 2005; Burnham & Hare 2007; Rigdon et al. 2009; Oda et al. 2011; Nettle et al. 2012a; although see Fehr & Schneider 2010). They are also less likely to take from others (Baillon et al. 2013), and more likely to donate their own resources to a charitable cause, at least under some conditions (Ekström 2011; Powell et al. 2012). Moreover, they are less likely to litter, more likely to contribute to an honesty box, and more careful following recycling rules (Bateson et al. 2006; Ernest-Jones et al. 2011; Francey & Bergmüller 2012). The eye images used as cues of conspecific observation in these studies are very varied and often not at all realistic (see e.g. Burnham & Hare 2007; Rigdon et al. 2009; Powell et al. 2012). People do not report noticing the eyes or feeling less anonymous at the conscious level (see Oda et al. 2011; Francey & Bergmüller 2012; Nettle et al. 2012a). This suggests that humans possess potent, automatic, easily evoked psychological mechanisms that modulate behaviour when conspecifics are watching. The functional significance of such mechanisms is presumably that conspecifics have the capacity to punish, or to use or spread negative reputational information, if they observe behaviours antithetical to their interests.

The phylogenetic origins of the mechanisms underlying the watching eyes effect have not yet been investigated, but they could be shared with other great ape species. Nonhuman primates show evidence of specialized psychological mechanisms for face processing that work in similar ways to those found in humans (Parr et al. 1998; Taubert & Parr 2012). Chimpanzees, *Pan troglodytes*, have a bias towards attending to faces more than other components in visual scenes, as humans do, although the bias is not as strong in chimpanzees (Kano & Tomonaga 2009), and the sequential fixations on the eye region of faces that are characteristic of humans are absent (Kano & Tomonaga 2010). Chimpanzees live in societies organized into dominance hierarchies that predict access to mates and food (Nishida 1979; Goodall 1986). The behaviour of chimpanzees indicates that they are acutely aware of their own position in the hierarchy (reviewed in de Waal 1986) as well as rank relations between others (Slocombe & Zuberbühler 2007). Subordinate chimpanzees are sensitive to whether a dominant can see a particular food item in their choice of whether to take it or not (Hare et al. 2000). This is due to the fact that dominants win out in scramble competition, and dominants may also punish subordinates for taking food items ahead of them (Jensen et al. 2007). Thus, there are reasons for expecting that there could be homologues of the watching eyes effect in chimpanzees.

Although the potential for third-party punishment may exist in some circumstances (e.g. von Rohr et al. 2012; but see Riedl et al. 2012; Engelmann et al. 2012), the data suggest that the predominant social risk for chimpanzees stems from direct second-party punishment rather than the broader suite of social and reputational consequences seen in humans. Thus, if there is a watching eyes effect in chimpanzees, it is most likely to be detectable in contexts in which the watching eyes represent cues that there might be a dominant individual nearby who is directly affected by the focal individual's actions, and who might administer second-party punishment.

Thus, to investigate a potential watching eyes effect, we devised a paradigm in which chimpanzees could take a food resource from the vicinity of either a larger-than-life stylized image of a chimpanzee face or an appropriate control image. We used stylized images rather than realistic ones to parallel the human experiments, in which highly stylized stimuli have been used. Our stimuli were black-and-white high-contrast renderings of a real photograph of a chimpanzee, similar to, although somewhat more detailed than, the Mooney faces used in human face perception research (Mooney 1957). Chimpanzees are known to recognize Mooney faces of chimpanzees as faces (Taubert & Parr 2012).

## Experiment 1

We investigated the impact of displaying either a larger-than-life chimpanzee face or a matched control image on chimpanzees' latencies to take food items from an array containing items of both high value (peanuts) and lower value (peanut-sized pieces of carrot). Given that taking food in plain sight of a dominant individual can lead to punishment, we predicted that in the face conditions, individuals would be more hesitant to take the food items, as reflected in longer latencies. We also predicted the increase in latencies would be particularly marked for individuals who are subordinate within their social groups.

We additionally predicted that in the face conditions, chimpanzees might switch from starting with the normally preferred food item (peanuts) to the less valued option (carrots), on the basis that taking a high-valued item from in front of an unfamiliar conspecific is a riskier behaviour than taking a low-valued item. Again, we expected this to be particularly true of individuals of low rank. Our array was asymmetrical, with one end closer to the face or control image than the other. If our subjects interpreted the face image as a watching conspecific, we expected that they would prefer to take items from further away from the image in the face as compared to the control conditions. However, this might interact with dominance. Where dominant chimpanzees have access to two food items, one also accessible to a subordinate, and one only accessible to themselves, they tend to choose first the one accessible to both parties, so that they will end up with both items (Hare et al. 2000). Subordinates instead avoid the item also accessible to the dominant. Thus, here, we predicted that dominant individuals in the face condition might shift their preference towards starting with the items closest to the face image, so that they could secure these before moving to the proximal parts of the array. As a check for whether our face stimuli were noticed, we also recorded time spent looking towards the stimulus during each trial.

### Methods

#### Subjects

Subjects were eight adult chimpanzees (four male, four female) from the same social group at Chimfunshi Wildlife Orphanage Trust, Zambia. These animals live in a large forested enclosure in a seminatural social group, but are habituated to humans, and are used to entering a building adjoining their enclosure for provisioning once a day. In the current experiment, individual chimpanzees voluntarily entered the building and remained inside for experimental sessions lasting up to 1 h before being released back into their enclosure. The dominance ranking of the eight individuals was assessed by K.A.C. on the basis of her longstanding experience working with these chimpanzees and independent interviews with the keepers who look after them. K.A.C. was blind to the results of the experiment when she provided her assessment. There was one pair of tied ranks. In this sample, all males outranked all females.

Both this experiment and experiment 2 were approved by Newcastle University Ethics Committee.

#### Experimental set-up

**Figure 1:**
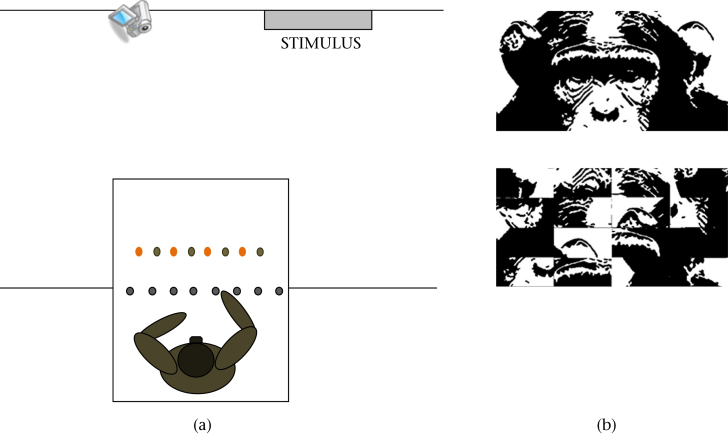
(a) Diagram of the experimental set-up for experiment 1, and (b) examples of face and control stimuli for experiment 1.

At the front of the experimental room was a concrete table 0.95 m high, half inside the room and half beyond a barred window onto a corridor whose gaps were sufficient for a chimpanzee to put a hand through. This allowed the subject to sit on the inner half of the table and reach through to take food items placed on the outer half by the experimenter (see Fig. 1). Experimental stimuli were displayed outside the experimental room on the wall facing the barred window at a distance of approximately 2 m and height of 1.75 m, offset to the right of the centre of the window from the chimpanzee's perspective. Trials were video recorded from a tripod-mounted camera at the same distance as the experimental stimuli but offset to the left.

#### Stimuli

To discourage habituation with repeated presentation, we created three different versions of a black-and-white cartoon-like chimpanzee face in Adobe Photoshop, using a stock photograph of a chimpanzee face as the starting point. One version, henceforth the small stimulus, featured just the upper face (eyes, nose and top of head) and measured 46 cm wide and 23 cm high, with an interpupillary distance (IPD) of 9 cm. The second version (large stimulus) was identical but measured 35 cm wide and 70 cm high (IPD = 14 cm). The third version (full stimulus) also included the muzzle and measured 43 cm wide and 35 cm high (IPD = 7.5 cm). The age and sex of the individual in the source image are not known. We sought to create apparent dominance by the image being slightly larger than life. For our full stimulus, implied bizygomatic breadth was 155 mm compared to actual male mean of 131.5 mm for chimpanzees from the Taï forest (Zihlman et al. 2008). For each stimulus, we created a control image by digitally cutting the image into 16 equal rectangles and inverting and shuffling these (see Fig. 1 for stimuli). Face and control stimuli were printed on durable fabric and attached to the wall using Velcro fastenings.

#### Experimental procedure

Prior to each trial, we laid out four shelled peanuts and four pieces of carrot of similar size to the peanuts, spaced 5 cm apart in an alternating line parallel to the barred window and 8 cm on the experimenters' side of it. The item closest to the experimental stimulus was always a peanut. Chimpanzees waited between trials in an antechamber from which the food items on the table were visible but, owing to the sightlines, the stimuli were not. At the beginning of the trial, the subject was admitted to the experimental room through a steel sliding door, which remained open during the trial allowing the chimpanzee to leave the experimental room at any point. Experimenters were out of sight during the trial. The trial was stopped 2 min after the door was opened. Following the trial the chimpanzee was encouraged to return to the antechamber and the door was closed. The chimpanzee was either released into the outdoor enclosure or retained for another trial. The intertrial interval for an individual chimpanzee was a minimum of 10 min but sometimes up to 3 days.

To check that the chimpanzees would enter the room and take items through the bars, and to establish that all individuals would consume both foods but valued peanut more highly than carrot, each subject had at least two practice trials with no stimulus displayed. Subjects proceeded to the experimental trials when a number of criteria were met. They had to take at least four items on two consecutive trials, and show a preference for peanuts as evidenced by taking all four peanuts before any carrot on one trial, or three peanuts before at least three carrots on two consecutive trials.

#### Design

The experiment was a within-subjects design; all subjects saw all three face–control pairs over six trials. Face–control pairs were presented in consecutive trials, but the order of face and control, and the order of the three pairs (small, large, full), were counterbalanced across subjects.

#### Data collection and analysis

Using the videos of the trials, we coded the latency from entering the room to taking each peanut and each carrot. We averaged across the four peanut latencies to produce the MeanLatPeanut variable, which is the mean latency to take a peanut on a given trial, and the MeanLatCarrot variable, which is the mean latency to take a carrot on a given trial. When the animal did not take a food item, the latency was scored as the duration of the trial. We also recorded the spatial position of the first two items taken, scored such that 1 represents the item closest to the stimulus and 8 the item furthest away. The MeanPosition variable represents the mean position of the first and second items taken.

To investigate the extent to which the chimpanzees attended to the experimental stimuli, we used JWatcher 1.0 (Blumstein et al. 2006) to code the proportion of visible time within the first minute of the trial the subject spent looking in the direction of the experimental stimulus. D.N. and M.B. independently coded all 48 videos several weeks after data collection and were blind to condition (the experimental stimulus was not visible in the videos), with video running at 0.5 speed. Gaze direction was difficult to assess precisely because of light contrast between the experimental room and the outside, and the lack of white sclera in chimpanzees. We therefore coded the chimpanzee as looking at the stimulus any time when its head direction was such that it could have been looking at the stimulus. The correlation between the two coders' scores was 0.91, and we therefore used the mean of the two scores as our Looking variable. Looking was positively skewed and hence was square-root transformed for analysis.

We used repeated measures general linear models (GLM) in SPSS version 19.0 (SPSS Inc., Chicago, IL, U.S.A.) with stimulus type (face or control) and size (small, large or full) as within-subjects factors and dominance rank as a between-subjects covariate to predict, successively, MeanLatPeanut, MeanLatCarrot, MeanPosition and Looking. Where sphericity assumptions were violated, reported significance tests are based on the Greenhouse–Geisser correction. We adopted an alpha level of 0.05 throughout, but cases where *P* ≤ 0.10 are discussed as nonsignificant trends.

### Results

**Figure 2:**
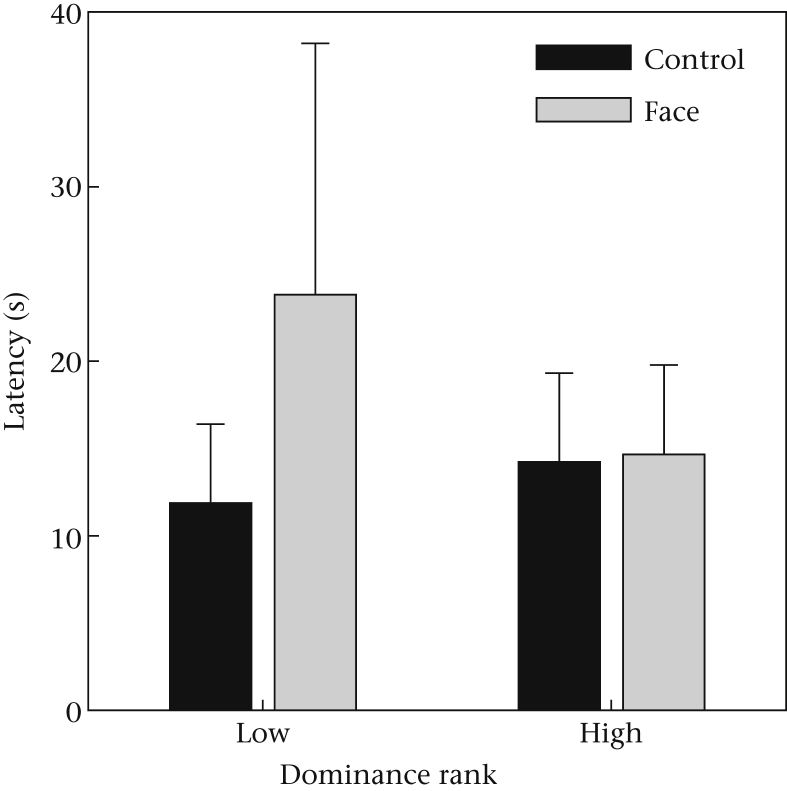
Estimated marginal mean latency to take peanuts in face versus control conditions, for the four lower- and the four higher-ranking individuals in experiment 1. Error bars represent one SE.

Data from the experiment are available as Supplementary material. All eight chimpanzees consumed peanuts in the experiment (seven of them on 6/6 trials and one on 5/6). MeanLatPeanut was greater in face than in control conditions (overall means ± SD: face = 19.22 ± 35.17 s; control = 13.05 ± 11.64 s). However, this difference was not significant in the repeated measures GLM model (effect of stimulus type: *F*_1,6_ = 1.19, *P* = 0.32). Size was not a significant predictor (*F*_2,14_ = 0.47, *P* = 0.64), and neither was the size*type interaction (*F*_1.1,6.6_ = 1.49, *P* = 0.27). There was a nonsignificant trend towards a stimulus*rank interaction (*F*_1,6_ = 3.82, *P* = 0.10). This was driven by the lower-ranking individuals (the four females in this sample) showing longer MeanLatPeanut in face versus control conditions, whereas no such trend was evident for the four higher-ranking (male) individuals (Fig. 2). The main effect of rank (*F*_1,6_ = 0.25, *P* = 0.63), and all other interactions, did not approach statistical significance.

As expected, chimpanzees were slower to take the carrots than the peanuts (paired *t* test with trial as the unit of replication: *t*_47_ = 6.98, *P* < 0.001; six of eight chimpanzees showed significantly longer MeanLatCarrot than MeanLatPeanut when considered singly, with the other two showing nonsignificant trends in the same direction; two chimpanzees did not consume any carrots at all). However, MeanLatCarrot was not significantly different between face (mean ± SD = 87.40 ± 71.82 s) and control (88.34 ± 71.73 s) conditions (*F*_1,6_ = 0.03, *P* = 0.86). There was a significant effect of stimulus size (*F*_2,12_ = 8.00, *P* < 0.01) and a significant size*dominance interaction (*F*_2,12_ = 6.29, *P* = 0.01). These effects were driven by subjects, and especially those of low rank, being slower to take carrots when the stimulus was large, regardless of whether it was a face or control image. The main effect of rank was not significant (*F*_1,6_ = 0.49, *P* = 0.51), and all other interactions were nonsignificant.

**Figure 3:**
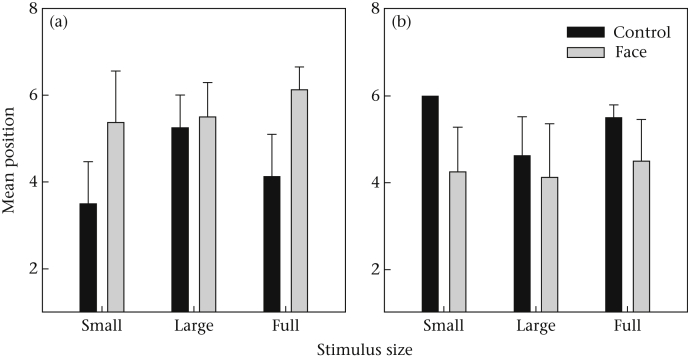
Estimated marginal mean of the position on the array of the first two items chosen, by stimulus size and type, for (a) the four lower-ranking individuals and (b) the four higher-ranking individuals in experiment 1. Error bars represent one SE.

MeanPosition was not significantly affected by stimulus type (means ± SD: face = 4.98 ± 1.89 s; control = 4.83 ± 1.57 s; *F*_1,6_ = 1.13, *P* = 0.33) or by stimulus size (*F*_2,12_ = 0.12, *P* = 0.89). There was a nonsignificant trend for a stimulus type*size interaction (*F*_2,14_ = 3.25, *P* = 0.08). This was due to the subjects choosing an item further from the stimulus in the full control than in full face condition. The main effect of rank was not significant (*F*_1,6_ = 0.07, *P* = 0.80), but there was a significant stimulus type*size*rank interaction (*F*_2,12_ = 4.56, *P* = 0.03). This effect was due to the low-ranking subjects starting with positions further from the stimulus in the face than control conditions, while the high-ranking subjects started with positions closer to the stimulus in the face than the control conditions, especially when the stimulus size was small or full rather than large (Fig. 3).

Subjects spent a larger percentage of time looking in the face (untransformed mean ± SD = 10.2 ± 9.2%) than control (5.8 ± 4.5%) conditions. However, this difference was not significant (*F*_1,6_ = 0.90, *P* = 0.38), and neither were the effect of stimulus size (*F*_2,12_ = 0.89, *P* = 0.44) nor the size*type interaction (*F*_2,12_ = 0.62, *P* = 0.56). When we added rank to the model, there was no main effect of rank (*F*_1,6_ = 0.93, *P* = 0.37), and no other main effects or interactions approached statistical significance.

### Discussion

Experiment 1 did not produce clear evidence that the chimpanzees were affected by the face stimuli while taking items from the array. However, there were a number of potentially interesting trends. There was a near-significant trend for the subordinate individuals to be slower to take the peanuts in the face conditions than in the control conditions, whereas the more dominant individuals showed no such pattern (Fig. 2). This trend is consistent with our predictions concerning the particular sensitivity of subordinates to the possibility of punishment and thus to cues of being watched by a nearby conspecific. However, given the nonsignificance of the effect, it does not constitute clear evidence that the prediction was supported.

There was also a significant interaction between stimulus type, stimulus size and dominance in predicting where in the array the animal would begin taking the items. Subordinate individuals began their consumption further from the stimulus in the face conditions, especially where the stimulus was small or full. Dominant individuals by contrast began closer to the stimulus in face conditions, again especially where the stimulus was small or full (Fig. 3). If the males in this study are perceiving themselves to be dominant to the stimulus whereas the females are not, then this is consistent with the previously observed pattern that subordinate individuals will avoid a resource also accessible to a dominant, preferring those accessible to themselves alone. By contrast, dominants when faced with the choice will often begin with the item accessible to both animals, in order to secure that one before moving on to the one accessible by themselves alone (Hare et al. 2000).

The other measures showed no evidence of a response to the face stimuli. Subjects looked longer on average at the face images than the control images, consistent with our prediction that these images would generate greater interest, but not significantly so, and this longer looking time did not translate into a significant increase in the latency to take the carrots. Properly controlling for multiple comparisons (we had four outcome measures and tested separately for a response on each) would mean that, overall, there is no robust evidence of any impact of the face stimuli. However, since some of our trends were consistent with our predictions, we designed a second experiment to investigate these further and establish whether they were reliable.

## Experiment 2

We designed experiment 2 to test whether the trends observed in experiment 1 were reliable by improving the design of experiment 1 and making the face stimuli more salient. Since there was no evidence of any experimental effect on latency to eat carrots, we simplified the array to consist of just the four peanuts. We also placed the food items further from the bars so that subjects would have to reach more markedly towards the experimental stimuli to take them. We used just the small stimulus and control from experiment 1, but enhanced them with coloured eyes. We also introduced a condition with the experimental stimulus much closer to the subject, at approximately a chimpanzee arm's length. Our predictions were for an increased latency to take the peanuts for subordinate individuals in the face conditions, and for subordinate individuals to take their first items further from the stimulus in the face than control conditions, with dominant individuals showing the opposite patterns. We expected that increased proximity of the experimental stimuli might enhance these patterns.

### Methods

#### Subjects

Subjects were 11 chimpanzees (five adult males, five adult females, one subadult male), comprising four adult males and three adult females who had participated in experiment 1, plus four new animals. Experiment 2 began 1 week after the end of data collection for experiment 1. Dominance information was again provided by K.A.C. blind to the results. As the subjects were from two different social groups, they could not all be assigned dominance relationships relative to one another. Instead, we assigned individuals a comparable score by dividing their position within their social group by the number of animals in that group (13 or 14). This yields an index of 1/*n* for an individual who is the most dominant within a group of *n* animals, through to 1 for an individual who is subordinate to all others in its group. For the seven individuals also in experiment 1, this index was perfectly correlated with the simple ranking used in the analysis for that experiment.

#### Experimental set-up

The set-up was the same as for experiment 1, except that rather than being displayed on the wall, the experimental stimuli were mounted on a freestanding board that could be moved. In the ‘far’ conditions, the board was placed so that the stimulus was 1.7 m from the subject, whereas in the ‘near’ conditions, the stimulus was only 0.7 m distant. In all conditions, the top of the stimulus was 1.75 m from the ground and offset to the right of the food array from the chimp's perspective.

#### Stimuli

**Figure 4:**
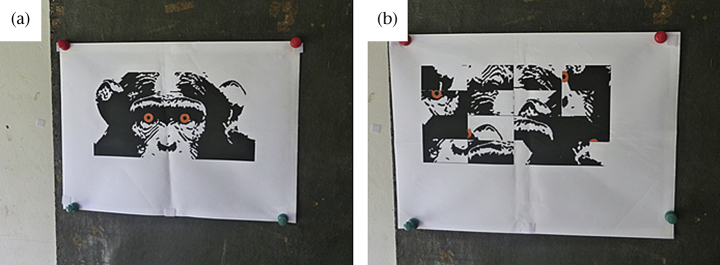
The (a) face and (b) control stimuli for experiment 2 in situ.

We used the small face stimulus from experiment 1, enhanced by the addition of orange discs for eyes with dark filled circles representing the pupils (Fig. 4). The control stimulus consisted of the small control stimulus from experiment 1, with two orange disks cut up and placed randomly in pieces on it (Fig. 4).

#### Procedure

Prior to the subject's entry, we mounted the experimental stimulus, and placed four shelled peanuts at 10 cm intervals in a line parallel to the barred window and 20 cm on the experimenters' side of it. As before, subjects were admitted into the experimental room through a door at the beginning of the trial. Trials were videoed with experimenters out of sight, and stopped after 2 min.

#### Design

The experiment was a 2 × 2 (distance × stimulus) within-subjects design, with each subject experiencing four trials (near face, near control, far face, far control). The orders of near and far and face and control were counterbalanced across subjects.

#### Data collection and analysis

We recorded the latency from entering the room to taking each peanut, and averaged these four latencies within a trial to produce MeanLatPeanut. We also scored the spatial position of the first item taken (Position1) from 1 (closest to the stimulus) to 4 (furthest). As for experiment 1, we used JWatcher to blind-code the amount of time spent looking in the direction of the experimental stimulus. Independent codings by D.N. and M.B. were correlated at *r* = 0.79 and their square-root-transformed mean was used for analysis. There were thus three outcome variables, MeanLatPeanut, Position1 and Looking. As for experiment 1, we conducted repeated measures GLMs with stimulus type and distance as the within-subjects variables, and dominance rank as a between-subjects covariate.

### Results

**Figure 5:**
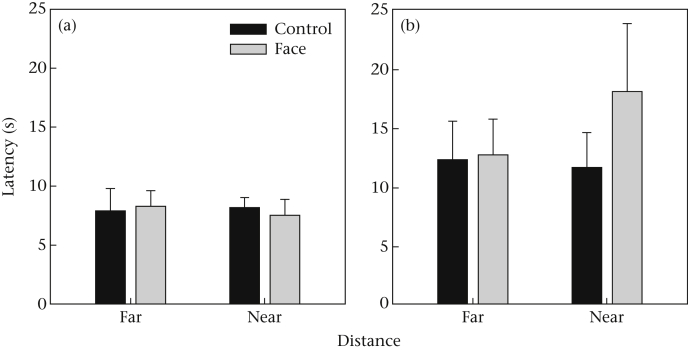
Estimated marginal mean latency to take peanuts by stimulus type and distance, for the (a) six lower-ranking and (b) five higher-ranking individuals in experiment 2. Error bars represent one SE.

Data from the experiment are available as Supplementary material. All chimpanzees consumed all peanuts on every trial. MeanLatPeanut was similar in the face (mean ± SD = 11.11 + 7.94 s) and control (10.12 ± 5.42 s) conditions. The GLM revealed no main effect of stimulus type (*F*_1,9_ = 0.21, *P* = 0.65) or distance (*F*_1,9_ = 0.91, *P* = 0.20). There was a nonsignificant trend for a stimulus type*distance interaction (*F*_1,9_ = 3.44, *P* = 0.10). This was driven by a tendency for MeanLatPeanut to be longer in the face than in the control conditions for the near stimuli only (Fig. 5; note in the figure the pattern is evident only for the higher-ranking half of the sample). The main effect of dominance rank (*F*_1,9_ = 2.71, *P* = 0.13) and all its interactions did not approach statistical significance, despite the qualitative pattern shown in Fig. 5.

Position1 was similar across face and control conditions (mean ± SD: face = 2.82 ± 1.18; control = 2.95 ± 1.17). In the GLM, there were no significant effects of stimulus type (*F*_1,9_ = 0.58, *P* = 0.47), distance (*F*_1,9_ = 0.27, *P* = 0.61) or any type*distance interaction (*F*_1,9_ = 0.09, *P* = 0.77). Neither dominance rank (*F*_1,9_ = 0.78, *P* = 0.40) nor any of its two-way interactions were significant. There was no evidence of any three-way interaction (*F*_1,9_ = 0.01, *P* = 0.97).

Looking was slightly higher in face (untransformed mean ± SD = 31.4 ± 14.4%) than control (29.9 ± 14.9%) conditions. However, neither the main effects of stimulus type (*F*_1,9_ = 0.01, *P* = 0.93) and distance (*F*_1,9_ = 0.02, *P* = 0.89) nor their interaction (*F*_1,9_ = 2.63, *P* = 0.14) approached statistical significance. Neither the main effect of dominance rank (*F*_1,9_ = 0.26, *P* = 0.62) nor any of its interactions approached statistical significance.

### Discussion

Our second experiment was intended to amplify the chances of finding significant responses to the face stimuli by enhancing the stimuli, moving them closer to the chimpanzee in one condition, and requiring the chimpanzee to reach further towards the experimental stimuli to obtain the peanuts. We also boosted our power by testing more animals. Despite these changes, we again found no clear evidence that the chimpanzees responded to the face stimuli differently from the control stimuli. There was only one trend at *P* < 0.10: in the near conditions, chimpanzees tended to be slower to take the peanuts when the stimulus was a face rather than a control.

The noteworthy trends and one significant result from experiment 1 did not recur in the second experiment. In experiment 1, lower-ranking individuals tended to be slowed down by face stimuli whereas higher-ranking ones were not. There was no evidence of any such interaction here, and in fact the qualitative pattern was for the higher-ranking individuals to be the ones slowed down by the near faces in experiment 2, as shown in Fig. 5. In experiment 1, lower-ranking individuals began with an item further from the stimulus in face conditions, whereas this pattern was reversed for higher-ranking individuals. There was no evidence of any such interaction in experiment 2.

## General discussion

We conducted two experiments on the willingness of chimpanzees to take food items close to an image of a watching conspecific as compared to a control image. Overall, we found no clear, repeatable effects of the face stimuli on behaviour. Although the chimpanzees looked towards the face stimuli more than the control stimuli on average in both experiments, these differences were not significant. There were no overall significant effects on latency to take the food items, the timings of taking higher-value or lower-value food types (experiment 1), or the proximity to the stimulus of the position in the array where the chimpanzee began.

There were a number of trends in the results of the experiments that were in line with our predictions. Specifically, in experiment 1, low-ranking individuals were differentially slower to take peanuts in the face than the control conditions, while in experiment 2, chimpanzees overall were slower in face than control conditions when the stimuli were placed very nearby. In experiment 1, high-ranking individuals began their consumption closer to the stimulus when it was a face image, whereas low-ranking individuals began further away from a face than a control image, at least for some stimulus sizes. However, this pattern failed to reappear in experiment 2.

Given that these trends mostly did not attain conventional statistical significance (and this would be even more true if we corrected for having tested several outcome variables in each experiment), and given that the same patterns did not appear in the two experiments, our overall conclusion is that we failed to detect any reliable response of these chimpanzees to these face images in this context. Failure to find an effect in a small sample does not of course mean that the true effect size is zero; there could be an effect which we had insufficient power to detect. Our sample size was constrained by the number of individuals who chose to enter the experimental area and complete the experiment. However, within-subjects designs are relatively powerful, as consistent between-subject variation in behaviour is controlled for. We used G*Power 3.1.7 (Faul et al. 2007) to calculate post hoc power for detection of a main effect of face stimulus given our sample sizes, number of trials and observed within-subject autocorrelations of the order of 0.5. To detect what is conventionally regarded as a large effect (*f* = 0.4; Cohen 1988), our power was 0.81 in experiment 1 and 0.85 in experiment 2. This is regarded as adequate power (Cohen 1988). Our power to detect a medium effect size (*f* = 0.25) was only 0.37 in experiment 1 and 0.43 in experiment 2. Thus, although we cannot conclude that the face stimuli had no effect, we had sufficient power to conclude that their effect was not large.

This stands in contrast to the human literature, in which substantial impacts of conspecific ‘watching eyes’ on behaviour have been very widely observed (see Introduction), often with large effect sizes (see e.g. Bateson et al. 2006; Powell et al. 2012). It is thus possible that there are genuine psychological differences between the two species in responsiveness to cues of conspecific attention. This would be consistent with a number of other findings. When viewing images, chimpanzees do attend to faces more than control regions of the image, but this bias is not as strong as it is for humans (Kano & Tomonaga 2009), and in particular, the repeated fixations on the eye regions typical of nonautistic humans are not seen in chimpanzees (Kano & Tomonaga 2010). While there is some evidence that, like humans, chimpanzees can modulate their behaviour according to the rank of a conspecific observer who might steal food or inflict immediate punishment (Hare et al. 2000; Jensen et al. 2007), unlike humans, there is currently no clear evidence that chimpanzees modulate their social behaviour in the presence of third-party observers for future benefit (Engelmann et al. 2012). If this is indeed the case, it would be a congruent conclusion that they might also be less responsive than humans to subtle cues of observation.

The evolutionary origins of these possible cognitive and behavioural differences presumably reside in the fact that humans are constantly exposed to the possibility of conspecific punishment and are constantly managing their reputation in the presence of conspecifics. More generally, human societies involve repeated instances of partner choice for collective action based on reputation; reputations can become widespread through gossip and language, and supported by normative cultural expectations for which there are sanctions for violation. Thus, for humans, there are potentially disastrous knock-on consequences for being observed doing the wrong thing. Chimpanzees do take coordinated social action, and have important and long-lasting social relationships. However, the intensity of social interdependence and social constraints is likely to be much lower than for humans, and cultural norms and expectations do not exist in the same way as they do for humans. For chimpanzees, there is a danger of immediate second-party punishment, but the consequences are perhaps not as pervasive as they would be for an observed human where there could be additional consequences for reputation. This might be an adaptive basis for greater sensitivity to cues of being watched in humans compared with chimpanzees.

An obvious objection to these conclusions is that we have only studied one, rather unusual population of chimpanzees, one experimental context, and only one set of conspecific face images. The study animals are habituated to human presence and although all but one of our subjects was human-reared, they have lived in large, seminatural enclosures with conspecifics for many years. The chimpanzees have some previous experience of participating in experiments, and are used to seeing humans and occasionally other chimpanzees through fences. They are also fed daily from the concrete table we used in our experiment. Thus, they may have learned that primates on the other side of fences can be safely ignored, and that any food on the tables is theirs to eat. We chose our foraging-like experimental scenario to simulate scramble competition during group foraging, as this seemed a reasonable context within which responses to conspecific observation might emerge. It is of course possible that such responses would be clearly seen in a different context. The stimuli we used were based on a real photograph of a chimpanzee, were of a general type that chimpanzees are known to respond to as faces (Taubert & Parr 2012), and were instantly chimpanzee-like to us. However, we cannot infer that there would not be a more marked response to different stimuli. Careful experimentation with different types of image would be needed to investigate this issue.

These limitations, although real, further highlight the difference between the human and chimpanzee evidence. In the human studies, effects have not been found in every context (Fehr & Schneider 2010), but they have been observed in many different populations and settings, including university students in the lab (Haley & Fessler 2005), neuroscientists on coffee break (Bateson et al. 2006), shoppers (Powell et al. 2012), bus-riders in a Swiss city (Francey & Bergmüller 2012) and even bicycle thieves (Nettle et al. 2012b). This suggests that the mechanisms responsible are widely available and not difficult to engage. The stimuli used have been extremely varied, for example in size, and often highly unrealistic, including stylized drawn backgrounds (Haley & Fessler 2005), small cartoons (Powell et al. 2012), a humanoid robot (Burnham & Hare 2007) and even three dots (Rigdon et al. 2009). In many of the experimental set-ups, it is absolutely clear that the stimulus could not possibly be a real observer and that there could be no real consequences, and yet the effects have still been found. Thus, although replication of these experiments with a wider range of chimpanzee-like face stimuli is needed, the fact that we could not find any strong effect with very salient stimuli of a general type chimpanzees are known to recognize as faces (Taubert & Parr 2012) does represent a contrast to the human literature. This contrast could well reflect genuine cognitive differences between the two species.
