# Supplementary material for: Responses of chimpanzees to cues of conspecific observation
Source: Anim Behav. 2013 Sep;86(3):595–602. doi: 10.1016/j.anbehav.2013.06.015 (PMC3763378; doi:10.1016/j.anbehav.2013.06.015)
Supplement: Supplementary file 3 [file mmc3.docx]

**Data supplement: explanatory notes**

**File ‘experiment1data.csv’**

| Trial | Number of the trial in the overall sequence of the experiment (numbering does not start at 1 as practice trials with no stimulus preceded these) |
| --- | --- |
| Date | Date in December 2012 |
| Time | A for AM or P for PM. AM trials were conducted between 0700 and 1100 and PM trials between 1500 and 1730 hours |
| Chimp | Name of chimpanzee |
| Rank | Rank of chimpanzee |
| Sex | Sex of chimpanzee |
| Stage | Trial number for the individual subject |
| Stimulus | Face or control stimulus |
| Size | Small, large or full stimulus |
| NoItems | Number of items consumed within the trial (of a possible eight) |
| PosItem1 | Position of first item taken |
| PosItem2 | Position of second item taken |
| MeanPos | Mean of PosItem1 and PosItem2 |
| LatP1-4 | Latency to take the first through fourth peanut |
| LatC1-4 | Latency to take the first through fourth carrot |
| MeanLatP | The mean (across peanuts) of the latency to take peanuts on that trial |
| MeanLatC | The mean (across carrots) of the latency to take carrots on that trial |
| Lookingcoder1 | Proportion of time looking at the stimulus, coder 1 |
| Lookingcoder2 | Proportion of time looking at the stimulus, coder 2 |
| Lookingaverage | Mean of Lookingcoder1 and Lookingcoder2 |
| Sqrtlooking | Looking average square-root transformed |

**File ‘experiment2data.csv’**

| Trial | Number of the trial in the overall sequence of the experiment (numbering does not start at 1 as practice trials with no stimulus preceded these) |
| --- | --- |
| Chimp | Name of chimpanzee |
| Rankindex | Rank index of chimpanzee |
| Sex | Sex of chimpanzee |
| Stimulus | Face or control stimulus |
| Distance | Stimulus near or far |
| Pos1 | Position of first item taken |
| Pos2 | Position of second item taken |
| Lookingcoder1 | Proportion of time looking at the stimulus, coder 1 |
| Lookingcoder2 | Proportion of time looking at the stimulus, coder 2 |
| Lookingaverage | Mean of Lookingcoder1 and Lookingcoder2 |
| Sqrtlooking | Looking average square-root transformed |
| LatP1-4 | Latency to take the first through fourth peanut |
| MeanLatPeanut | The mean (across peanuts) of the latency to take peanuts on that trial |
